# Accuracy of Deep Neural Network in Triaging Common Skin Diseases of Primary Care Attention

**DOI:** 10.3389/fmed.2021.670300

**Published:** 2021-08-26

**Authors:** Mara Giavina-Bianchi, Eduardo Cordioli, André P. dos Santos

**Affiliations:** Department of Telemedicine, Hospital Israelita Albert Einstein, São Paulo, Brazil

**Keywords:** primary care attention, common skin lesions, dermatology, deep neural network, articial intelligence

## Abstract

Access to dermatological care can be challenging in certain regions of the world. The triage process is usually conducted by primary care physicians; however, they may not be able to diagnose and assign the correct referral and level of priority for different dermatosis. The present research aimed to test different deep neural networks to obtain the highest level of accuracy for the following: (1) diagnosing groups of dermatoses; (2) correct referrals; and (3) the level of priority given to the referral compared to dermatologists. Using 140,446 images from a teledermatology project, previously labeled with the clinical diagnosis, and their respective referrals, namely biopsy, in-person dermatologist visits or monitoring the case via teledermatology along with the general physician, 27 different scenarios of neural networks were derived, and the algorithm accuracies in classifying different dermatosis, according to the group of the diagnosis they belong to, were calculated. The most accurate algorithm was then tested for accuracy in diagnosis, referral, and level of priority given to 6,945 cases. The GoogLeNet architecture, trained with 24,000 images and 1,000 epochs, using weight random initialization and learning rates of 10^−3^ was found to be the most accurate network, showing an accuracy of 89.72% for diagnosis, 96.03% for referrals and 92.54% for priority level in 6,975 image testing. Our study population, however, was confined to individuals with chronic skin conditions and, therefore, it has limited value as a triage tool because it has not been tested for acute conditions. Deep neural networks are accurate in triaging, correct referral and prioritizing common chronic skin diseases related to primary care attention. They can also help health-care systems optimize patients' access to dermatologists.

## Graphical Abstract

Graphical Abstract showing the research flow from the dataset base images, classification of the dermatoses according to categories, referral and level of priority; training and validation in different scenarios to the final test, using the algorithm with the best performance. 
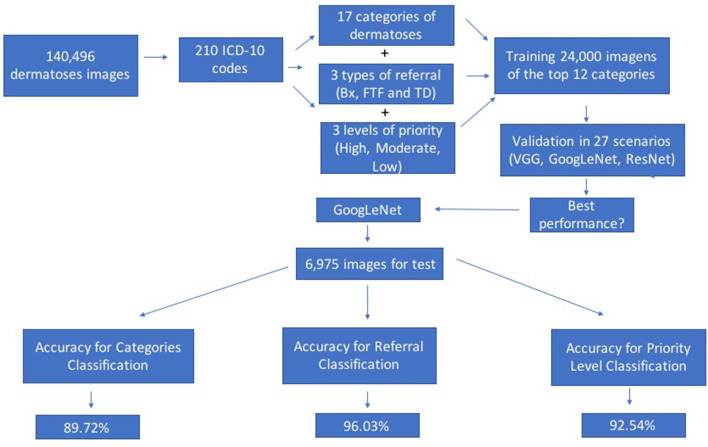

## Introduction

Health access is a serious challenge for most of the worldwide population. It becomes even more evident when the necessary assistance requires a specialized professional. In Brazil, the specialty of Dermatology was the second-most referred to by primary care physicians, amounting to 17.78% of all the referrals with the average waiting time being more than 100 days for an appointment (1, 2).

A large number of dermatologist referrals occur mainly due to the wide variety of dermatoses, which makes the diagnosis challenging for primary care physicians. A study conducted by the Brazilian Society of Dermatology indicated that, in 2018, only 9.1% Brazilian municipalities had specialists in dermatology (3). This heterogeneous distribution of the medical population

especially in remote areas, contributes to the increase in seeking means of care such as telemedicine. The effectiveness, accuracy, and reliability of dermatological diagnosis via telemedicine have been widely studied and are considered equivalent to those performed by dermatologists in face-to-face consultations (4–6).

The digital nature of these services, added to the recent developments in the field of image processing, provide an extremely fertile environment for research related to the application of artificial intelligence (AI) in the identification and diagnosis of skin lesions. In 2016, Shrivastava developed an algorithm based on support vector machine (SVM) capable of identifying psoriasis with 99.81% accuracy (7).

In 2018, in a research by Han et al., a classification algorithm built with deep neural networks showed 91% accuracy in the task of differentiating pigmented lesions between benign and malignant using a sample of 21,306 images (8). A large body of research in the literature points to a satisfactory accuracy of AI algorithms in identifying various types of skin diseases, ranging from simple onychomycosis (9) to melanomas (10–12).

However, previous studies generally focused on one disease or a limited group of diseases. To study the application of these technologies in the larger context of public health, a broader approach is needed to classify the dermatoses in established categories and suggest appropriate treatment approaches. In addition to focusing on the diagnosis of a single disease, it was observed that many studies have utilized image acquisition protocols and equipment designed specifically for AI in order to enhance the accuracy of classification algorithms. However, these image-acquisition protocols are too time consuming for health-care professionals to implement, and the equipment used are not available in most primary health-care units. This hinders the implementation of the algorithm in the public health system.

The authors identified an opportunity to evaluate the performance of these techniques in a broader context which does not classify lesions as belonging or not belonging to a specific diagnosis, rather in a more comprehensive classification, using simple protocols that can be applied by day-to-day primary health-care professionals.

The image acquisition equipment used in the present study are available among primary care networks. The purpose of the study was to optimize stages of the diagnostic process to provide health-care professionals with a diagnostic support tool that is capable of correctly and quickly screening and referring to cases to the specialist, according to their priority level.

The primary goal of the current work was to evaluate the accuracy of different algorithms of deep neural networks for triage and support diagnosis of skin lesions. The second aim was to test the accuracy of the algorithm for the best performance on the diagnosis, referral and level of priority, compared to those performed by dermatologists, using a test with clinical images *in silico*.

## Materials and Methods

### Acquisition of Clinical Images for the Development of the Neural Network

The present study was approved by the Ethics Committee of the Hospital Israelita Albert Einstein (project number: 3541-18). 140,446 images were utilized corresponding to 57,568 skin lesions, obtained between July 2017 and August 2018 from a philanthropic assistance project between Albert Einstein Israel-Brazilian Benevolent Society and the São Paulo City Hall. The project and its results have been described in detail previously (1).

Briefly, patients requiring primary care attention and waiting for an appointment with a dermatologist in the public health-care system of the city were directed to three municipal hospitals, where photos of their skin lesions were collected by health technicians using the default Samsung Galaxy S7 smartphone camera in conventional offices. These images, together with the demographic data and a brief clinical history, were uploaded to the Amazon Web Services platform and diagnosed by 13 dermatologists who had to choose among three possible referrals: (1) biopsy of the lesion; (2) a face-to-face visit to the dermatologist; or (3) suggesting the best conduct for the treatment and/or monitoring the cases via teledermatology along with the primary care physician. The 57,568 diagnosed lesions were classified into 210 International Classification of Diseases-10 (ICD-10) codes, which were grouped into 17 categories based on the nature of the dermatoses for better evaluation of the results. The categories are described in Table 1 and are the targets for predicting the diagnosis in the present study. The total number of images in each category, their corresponding percentage, the number of images used to test the algorithm and referrals with their respective priorities, according to our dermatologists' standards of care are shown in Table 1. The ICD-10 codes and the main diseases that make up each category are listed in Supplementary Table 1.

**Table 1:** Categories of target classes dermatological diseases, their contribution in the dataset of images, referral and level of priority, according to our dermatologists' standard of care.

	**Category**	**Total images dataset (*n*)**	**Total images dataset (%)**	**Test images (*n*)**	**Referral**	**Priority**
1	Benign tumor	31,998	22.78	1,600	TD	low
2	Eczema	25,217	17.95	1,261	TD	moderate
3	Pigmentation disorder	19,825	14.12	992	TD	low
4	Superficial infection/Infestation	18,963	13.50	949	TD	moderate
5	Inflammatory disorder	15,228	10.84	762	TD	moderate
6	Benign cyst	7,776	5.54	389	FTF	low
7	External cause	5,916	4.21	296	TD	low
8	Genetic cause	5,077	3.61	254	TD	low
9	Not grouped	2,474	1.76	123	TD	low
10	Metabolic cause	2,438	1.74	121	FTF	low
11	Malignant tumor	2,375	1.69	118	BIOPSY	high
12	Pre-malignant (actinic keratosis)	2,200	1.57	110	BIOPSY	high
13	Connective tissue disorder	371	0.26	0	FTF	high
14	Adverse drug reaction	339	0.24	0	TD	moderate
15	Deep/systemic infection	128	0.09	0	FTF	high
16	Bullous disease	61	0.04	0	TD	high
17	Factitial dermatitis	60	0.04	0	TD	low
18	Total	140,446	100%	6,975	–	–

*TD, teledermatology; FTF, face-to-face dermatologist*.

### Development of Artificial Neural Networks

To mitigate the problem of lack of focus on injuries, all the images were cut individually using Microsoft Paint so that only the region of interest related to the lesion was preserved for analysis. Following this, in order to standardize the network input, images were resized to 224 ×224 pixels. The errors obtained in the classification of the training set and validation were calculated using the mean square error equation (MSE). It was chosen among the various loss functions used for the classification field because it was the most frequent suppervisioned learning in medical literature at the time of the experiment. More technical information on algorithm pipeline is found in Supplementary Material 2.

The programming language used was Python; all neural network models were trained and tested on Keras API using Tensorflow as the backend in two Amazon instances: p 2.xlarge with a single Nvidia Tesla k80 GPU, g3.4xlarge with a single Nvidia Tesla M60 GPU.

### Experiments

Two initial exploratory experiments conducted previously to the following are presented in Supplementary Material 3.

#### First Experiment

To test the accuracy of different neural networks, 24,000 random images were utilized to train the 12 groups of most frequently identified dermatoses (Table 1), corresponding to 99.32% of the total volume of the diagnosed diseases. The 12 groups were composed of the classes: benign tumor, eczema, pigmentation disorder, superficial infection/infestation, inflammatory disorder, benign cyst, external cause, genetic cause, not grouped, metabolic cause, malignant tumor and pre-malignant. The category “Pre-malignant” had only 2,200 images and was the only one in which the same images were used both in the training and validation sets (Table 1).

Twenty seven scenarios were evaluated based on architectural variations, the initialization strategy, the weights, and the learning rates. The VGG, GoogLeNet and ResNet architectures were evaluated using weight initialization of fixed feature extractors (FFE), extension of the model and reinitialization of weights (EMRW), and random initialization (RI). We have chosen those architectures based on previous works (8, 9, 13), which showed interesting results with them. As the first experiment intended to be exploratory, we used different initialization techniques, including random initialization, which could be seen as not seen suitable for our dataset. IMAGEnet was the base assembly to apply for weight initialization. The learning rate value was adjusted between test scenarios. The parameter variations were 1e-03, 1e-06, and 1e-09, respectively.

The other parameters of the architectures followed the configuration presented in the literature (14). For training, momentum rate with a value of 0.9 and images of the dataset were processed in groups of 32 images simultaneously were used. The training was carried out over 1,000 epochs. To avoid data unbalance due to the disparity between the number of images in each group, the same number of images were used in each of the 12 groups for training and validation. For the validation, 200 random images, which were not present in the training set of each group, were used.

#### Second Experiment

During the second experiment, in the final test with the algorithm yielding the best performance among the deep neural networks, 5% of images from each category of dermatoses were used with 7.022 images belonging to the 17 groups, as shown in Table 1. The five groups presented at the bottom of the table (connective tissue disorder, adverse drug reaction, deep/systemic infection, bullous disease and factitial dermatitis), however, were disregarded due to the small number of cases, resulting in a total of 6,975 cases tested.

## Results

### First Experiment

Table 2 presents the results of 27 different scenarios tested in the first experiment to determine which neural network achieves the best performance.

**Table 2:** Results of the first experiment to test the accuracy of different neural networks according to architecture, weights and learning rate.

**Scenario**	**Architecture**	**weight Initialization**	**Learning rate**	**Training accuracy**	**Validation accuracy**	**Training error**	**Validation error**	**Validation specificity**	**Validation sensitivity**
**1**	**VGG**	**FFE**	**10** ^**−3**^	**99.97%**	**87%**	**0.0120**	**0.7359**	**96.76%**	**87.76%**
2	VGG	EMRW	10^−3^	76.61%	66.66%	0.5899	0.8782	91.50%	68.2%
3	VGG	RI	10^−3^	97.72%	86.45%	0.0722	0.7367	96.51%	86.6%
4	GoogLeNet	FFE	10^−3^	100%	88%	0.0002	0.6811	88.59%	97.01%
5	GoogLeNet	EMRW	10^−3^	92.46%	58.33%	0.1943	2,027	89.75%	62%
**6**	**GoogLeNet**	**RI**	**10** ^**−3**^	**98.09%**	**90.62%**	**0.0774**	**0.4928**	**97.73%**	**90.86%**
7	ResNet	FFE	10^−3^	19.53%	20%	1.6094	1.6094	80%	20%
8	ResNet	EMRW	10^−3^	98.55%	25%	0.0288	8,040	80%	20%
9	ResNet	RI	10^−3^	15.27%	14%	1.6094	1.6094	79%	16%
10	VGG	FFE	10^−6^	51.92%	60%	1,2061	1,181	89.95%	62.26%
11	VGG	EMRW	10^−6^	56.85%	51.04%	1.1603	1.2	87.98%	54.33%
12	VGG	RI	10^−6^	20%	26%	1.6094	1,6093	80%	20%
13	GoogLeNet	FFE	10^−6^	88.16%	84%	0.3768	0.4937	95.94%	85%
14	GoogLeNet	EMRW	10^−6^	66.21%	40.62%	0.899	1,66716	85.13%	43.3%
15	GoogLeNet	RI	10^−6^	43.28%	38.54%	1.3286	1.6240	84.65%	40.59%
16	ResNet	FFE	10^−6^	17.37%	26%	1.6109	1,608	80.26%	21%
17	ResNet	EMRW	10^−6^	79.77%	25%	0.5516	1,99700	80%	20%
18	ResNet	RI	10^−6^	34.87%	30%	1,44680	1.6094	82.74%	33.32%
19	VGG	FFE	10^−9^	20%	20%	1,77511	1,66637	80%	20%
20	VGG	EMRW	10^−9^	20.24%	20.83%	1.6809	1,66434	80%	20%
21	VGG	RI	10^−9^	20.76%	14.58%	1,694	1.6094	80%	20%
22	GoogLeNet	FFE	10^−9^	19.81%	25%	1.7363	1.6406	81.12%	25.2%
23	GoogLeNet	EMRW	10^−9^	18.26%	11.45%	1,66825	1.7704	77.47%	11.20%
24	GoogLeNet	RI	10^−9^	20.22%	16.66%	1,685	2,22818	79.50%	17.60%
25	ResNet	FFE	10^−9^	22.7%	19%	1.6269	0.04	79.75%	19%
26	ResNet	EMRW	10^−9^	18.85%	14%	2.1515	1.8448	79.69%	18.66%
27	ResNet	RI	10^−9^	19.42%	15%	1,833	2,33286	79.35%	16.79%

*FFE, fixed feature extractors; EMRW, extension of the model and reinitialization of weights; RI, random initialization*.

*The bold in lines for scenarios 1 and 6 of the this table, they were the best results obtained in the first experiment*.

In the training phase, the best accuracy was observed in scenarios with a learning rate of 10^−3^. All three architectures showed an accuracy of 98–100%. However, when verifying the values of the validation phase, the GoogLeNet architecture obtained the best performance out of all using the RI approach, with 90.62% accuracy.

### Second Experiment

The GoogLeNet network, using IR weight initialization was, then, tested in 6,945 images, obtaining an accuracy of 89.72%. Table 3 shows the confusion matrix in the diagnostic accuracy of the test in different categories of dermatoses.

**Table 3:** Confusion matrix of this diagnostic accuracy for GoogLeNet architecture.

	**Genetic cause**	**Metabolic cause**	**Traumatic cause**	**Benign cyst**	**Pigmentation disorder**	**Eczema**	**Superficial infection**	**Inflammation of unknown cause**	**Not grouped**	**Benign tumor**	**Malignant tumor**	**Pre-malignant tumor**
Genetic cause	236	1		9		53	9		3	38		
Metabolic cause		119	2			34	7	6	4		4	
External cause	1		272			3	5		1	14		2
Benign cyst	5			352	27	24	23	12	5			
Pigmentation disorder			4	7	898	36	29	20	5	11	2	
Eczema			2	2		1,041			3			
Superficial infection/infestation			13		29		859		2			
Inflammation disorder	3			9		2		690				
Not grouped	9		3	7	23	68		34	94		3	3
Benign tumor		1			11				1	1,488		0
Malignant tumor					4		15		2	20	108	4
Pre-malignant				3			2		3	29	1	101

*Values of the color shade are the true positive results*.

Observing the distribution of errors in Table 3, a regularity can be noticed in the number of errors and hits for each class except for “ungrouped,” which shows not only a smaller number of hits among the lesions but also in a greater number of classes classified as other lesions with this label.

An analysis was conducted of the referral and its priority proposed by the algorithm regardless of the lesion category. Regarding referrals, eight groups (6,237 images) indicated referral for teledermatology, two groups (510 images) for face-to-face dermatologist appointments and two groups (228 images) for biopsy. Among the 12 groups of lesions assessed in the test dataset, up to six had low priority (3,479 images), four had moderate service priority (3.268 images), and two had high priority calls (228 images). The distribution of the priority indication of the service and the routing suggested in the classification of the images are presented in Table 4.

**Table 4:** Confusion matrices for referrals and level of priority using GoogLeNet architecture.

		**Results predicted by A**I		
	**Referral**	**Teledermatology**	**Dermatotolgy**	**Biopsy**
Dermatologists' Choice (ground truth)	Teledermatology	6,013	36	10
	Dermatology	149	471	4
	Biopsy	75	3	214
	Total	6,237	510	228
	Priority	Low	Moderate	High
	Low	3,354	364	12
	Moderate	64	2,887	2
	High	61	17	214
	Total	3,479	3,268	228

The overall accuracy was 96.03% (6,013+471+214 = 6,698/6,975) for the referral of the patient and 92.54% (3,354+2,887+214 = 6,455/6,975) for the priority definition task. The referral confusion matrix showed equivalent specificity percentages in all possible outcomes, 96.41% (6,013/6,237) for referral to the teledermatologist, 92.35% (471/510) to attend dermatologist in person and 93.86% (214/228) for biopsy. As for the priority, it was noted that 93.86% (214/228) high priority lesions received a pertinent indication in the algorithm. However, 78 cases classified as high priority by the dermatologists were classified as low (61) and moderate (17), determining a specificity of 73.23% (214/292).

## Discussion and Conclusions

The present study tested 27 scenarios of deep-learning algorithms to determine the most efficient one for classifying common skin lesions in primary care attention into one of the predefined categories, reaching an accuracy of 90.6% in the validation phase and 89.7% in the test phase for 6,975 cases. These results were considered good compared to those in the literature. One of the studies in the literature aimed to use deep neural networks to classify 26 common dermatoses in primary care, with an overall accuracy of 0.66 in 963 cases (15). In another study, 3,501 cases were tested (using two different validation sets) for 134 classes of skin lesions, showing an accuracy of 56.7% in one test group and 44.8% in the other (16). In another research with 5,014 validated cases, AI obtained 76.9% accuracy in classifying 40 common dermatoses (17). Further, in a recently conducted study, 340 teledermatology images were tested for 174 different dermatological diseases, obtaining an accuracy of 41.2% (18).

Currently, a large number of articles show the development of algorithms for diagnostic support of diseases such as melanoma or a group of disorders such as skin cancer mainly using dermoscopic images (19–21). However, the use of dermoscopy may not be feasible on a large scale such as public health. For this reason, the present study used clinical images. Notably, the definitive diagnosis of malignant lesions to date is obtained by histopathology examination, i.e., through the microscopic analysis of the lesion in part or in totality after its surgical excision (biopsy). Therefore, although physicians or an algorithm may suggest skin cancer, the diagnosis is only confirmed by histopathology. Thus, if the biopsy referral is correct, this may be more significant than the diagnosis *per se*. An added advantage, especially in cases where the wait is sometimes as long as 6 months or more, can be if the priority of the disease is determined in addition to providing the correct referral. In cases of skin cancer, if the algorithm correctly points to high priority, it can benefit the patient as well as the health system as a whole. Thus, we consider our results with an accuracy of 96.0% for referrals and 92.5% for priority to be significant, as this data has not yet been reported in the literature.

Another fundamental aspect of the current study is the search for a simpler and low-cost means to obtain images: those clicked by health technicians using the standard photographic camera of a smartphone in regular offices. The use of common technology, with easy access and operability makes it viable to scale the present study. As the images and data were obtained by health technicians, the demand for the physician's specialized workforce is reduced, possibly also lowering costs in the public system.

In terms of the development of neural networks, several challenges were identified in the present study such as the labeling of skin lesions by dermatologists which was done through ICD-10. Initially, it was expected that the present work would be able to create a classifier that can identify the ICD of each lesion. However, after the reports were extracted, it was found that, in addition to the large number of classes (*n* = 210), some ICD codes had a limited number of copies—around two or three images—disabling the training of an algorithm for their identification. Another situation was the low number of cases (*n* = 2,200) in the premalignant category, for which the same images had to be used for both validation and testing. Although this situation is against the basic rules of deep learning algorithm development, we used the same images for validation and test datasets because we did not have enough number of cases in the pre-malignant class to divide it into the different datasets, as we had in the other classes in order to prevent imbalance among the classes.

Also, the category “not grouped” performed poorly in accuracy due to the greater heterogeneity of the images that make up this data set. This class encompassed images showing no lesions at times, or lesions which did not characterize a specific classification, and nail disorders that could not be specified in other groups (Supplementary Table 1). Thus, less similarity was observed between the images in the data set and, in some cases, with lesions of other classes.

There are limitations to our work. Our study population was confined to individuals with chronic skin conditions and, therefore, it has limited value as a triage tool because it has not been tested for acute conditions and might not not work in this setting. It is also important to note that the algorithm was in no way intended to take over the physician's role. Its objective lies only in the screening of chronic dermatological lesions, offering health professionals a supporting tool in the confirmation of clinical diagnosis and increasing their productivity in the evaluation of patients. This can optimize medical access for the more severe, surgical, or complex cases and direct them to the right referral and priority.

There are several future possibilities based on the present study. For clinical research, the most obvious pathway will be to apply the algorithm in real-life settings to compare its performance with that of physicians and assess its degree of accuracy. Second, in case of good performance, the use of the algorithm should be verified as this tool would modify medical management. In the field of computing, many possibilities are present for increasing the algorithm's accuracy: use of other network architectures such as ResNeXT, DenseNet and SE-Net; use of demographic data and clinical history of patients; and use of data augmentation for groups of dermatoses with less representation.

## Data Availability Statement

The raw data supporting the conclusions of this article will be made available by the authors, without undue reservation.

## Ethics Statement

The studies involving human participants were reviewed and approved by Ethics Committee of the Hospital Israelita Albert Einstein (project number: 3541-18). Written informed consent from the participants' legal guardian/next of kin was not required to participate in this study in accordance with the national legislation and the institutional requirements.

## Author Contributions

MG-B was responsible for study's design, data collection, and writing and reviewing the article. AS was responsible for study's design, data collection, and performing the experiments and reviewing. EC was responsible for study's design, data collection, and reviewing the article. All authors contributed to the article and approved the submitted version.

## Conflict of Interest

The authors declare that the research was conducted in the absence of any commercial or financial relationships that could be construed as a potential conflict of interest.

## Publisher's Note

All claims expressed in this article are solely those of the authors and do not necessarily represent those of their affiliated organizations, or those of the publisher, the editors and the reviewers. Any product that may be evaluated in this article, or claim that may be made by its manufacturer, is not guaranteed or endorsed by the publisher.
