# Supplementary material for: Accuracy of Deep Neural Network in Triaging Common Skin Diseases of Primary Care Attention
Source: Front Med (Lausanne). 2021 Aug 26;8:670300. doi: 10.3389/fmed.2021.670300 (PMC8427035; doi:10.3389/fmed.2021.670300)
Supplement: Supplementary file 1 [file Data_Sheet_1.docx]

**SUPPLEMENTARY FILES**

**S1** **Table: International Classification of Diseases (ICD) -10 codes included in each category of dermatoses:**

| **ICD-10 code** | **Dermatoses** |
| --- | --- |
| L00, L01.0, L02.9, L03.9, I88.9, L08.0, L08.1, L30.3, L75.0, B43.9, B85.2, B35.0, B35.2, B35.3, B35.4, B35.5, B35.6, B35.9, B02, B00, B36.0, B35.1, L08.8, B37.9, N48.1, B86, L03.0 | Superficial Infection |
| L05.0, L05.9, L72.0, L72.1, L72.2, L72.9, L72.8, D17.3 | Benign cyst |
| L10.0, L10.1, L10.2, L10.3, L10.4, L10.5, L11.1, L12.0, L12.1, L12.2, L12.3, L13.0 | Bullous Disease |
| L11.0, L57.4, L64.9, L66.9, L67, L68.3, L85.0, L85.1, L85.2, L90.6, L60.2, L60.5, E70.3, R61.9 | Genetic cause |
| L20.9, L21.9, L22, L23.9, L24.9, L28.0, L28.1, L29.9, L30.0, L30.1, L30.2, L30.4, L30.5, L56.2, L74.0, L74.1, L75.2, L85.3, I83.1, K13.0, L83.1 | Eczema |
| L27.0, L27.1, L43.2, L51.2, L51, L56.0, L56.1, L53.8 | Adverse drug reaction |
| L40.4, L40.1, L40.2, L40.3, L40.5, L41.0, L41.1, L41.2, L41.3, L41.4, L41.5, L42, L43.0, L43.1, L43.3, L44.0, L44.1, L44.2, L44.3, L44.4, L43.9, L50.1, L50.2, L50.3, L50.4, L50. 5, L50.6, L50.9, L52, P83.1, L53.2, L63.0, L63.1, L63.2, L63.9, L66.0, L66.1, L66.2, L66. 3, L70.0, L70.1, L70.4, L70.9, L71.9, L25.1, L73.0, L73.2, L81.7, L88, L92.2, L95.0, L95. 1, L95.9, L97, L98.2, L98.3, L98.4, E85.4, L53.1, L92.0 | Inflammatory disease of unknown cause |
| T30.1, T30.3, T30.2, L56.3, L56.4, L57.1, L57.2, L57.3, L57.5, L58.0, L58.1, L59.0, L73. 1, L83, L84, L89, L90.5, L90.8, T81.5, L60.0, L60.1, L60.3 | Traumatic cause |
| D04, L56.8 | Pre-malignant |
| L65.0, L65.1, L65.2, L65.9, L68.0, L87, L92.1, L60.4, E80.1, | Metabolic cause |
| L71.1, L82, L91.0, L98.0, B08.1, B07, D23.9, D18.0, D21.9, D22.9, Q82.8, A63.0, H02.6, D36. 1 | Benign tumor |
| L81.0, L81.1, L81.2, L81.3, L81.5, 81.4, L80 | Pigmentation disorder |
| L90.0, L93, L93.0, L93.1, L94.0, L94.1, L94.2, L94.4, L94.5, L94.6, M33.9 | Collagen disease |
| L98.1 | Psychiatric disorder |
| D04.9, C43.9, C80, C44.9, I78.1, Q82.5, C84.0 | Malignant tumor |
| A30.9, B42.9, B55.9, B41.9 | Deep infection |

**S2: Experimental Methodology**

The experimental methodology used in this research was divided into five topics: Data collection, which describes the process of obtaining images and evaluating experts; Class labeling, which explains how the study constituted the seventeen classes for use in predictive modeling; Tools used where each tool is individually described and how they were applied throughout the project; Test scenarios that run through the entire stage of preparing and training the algorithms.

**S2.1 Data Collection**

This project has available a dataset consisting of 140,446 images corresponding to 57,568 dermatological lesions. The images were obtained between July 2017 and August 2018 through a philanthropic assistance project, the result of a partnership between the Albert Einstein Israelita Beneficent Society and the city of São Paulo. The lesions were diagnosed by thirteen specialists and classified into 210 different ICD-10 codes (International Code of Diseases).

Those images were collected by nursing technicians equipped with a Samsung Galaxy S7 smartphone at five points in the city of São Paulo. The patients were residents of the city who were waiting for specialized care in the Unified Health System (SUS). For cases where the patient's lesion lacked a biopsy to confirm the diagnostic hypothesis, the report issued after the examination to confirm the diagnosis was considered; for the other cases, the lesion was referred for evaluation by a second specialist. Once there was a consensus between the opinions, the diagnosis was considered correct, otherwise, there was a divergence of opinion among the specialists, a third professional was consulted to assist in the classification of the lesion.

It is noteworthy that the objective of the project was to evaluate the feasibility of applying deep neural networks to perform the screening of skin lesions through images collected based on protocols and equipment available in the Unified Health System (SUS). The collection protocol is based on the capture of three images of the lesion, at first, 30 centimeters away, then 10 centimeters away, and the third image taken at a horizontal angle to allow the dermatologist to visualize the lesion volume. All photos were captured by a traditional smartphone camera. The quality of the images present in the dataset proved to be much lower when compared to the experiments that presented the best results in the literature. The dataset showed large variations in lighting conditions, presence of objects unrelated to the lesion in the images, variation in the distance between the device and the lesion over the different collections, lack of background standardization, poor camera framing at the time of image acquisition and lack of focus on the lesion.

To mitigate the problem of lack of focus on the lesions, all images were individually cropped by the Microsoft Paint tool so that only the region of interest related to the lesion was preserved. After cropping, to standardize the input of the network, the images were resized to the size of 224 x 224 pixels.

**S2.2 Image labeling and target class definition**

After the images collection, it was necessary to carry out a study to understand the representativeness, predominance and characteristics of the types of lesions present in the data set. Labeling of skin lesions by dermatologists was performed using the International Code of Diseases (ICD-10). Initially, it was expected that with this work it would be possible to create a classifier capable of identifying the ICD of each lesion. However, from an extraction of the reports, it was identified that, in addition to the high number of classes, some CIDs had a very limited number of copies, around two or three images, making it impossible to train an algorithm for their identification. Faced with this challenge, the scope of the project was adjusted to predict the patient's referral, suggest the priority of care and identify the patient's lesion group to support the diagnosis. Thus, the committee of dermatologists participating in the project grouped the 210 ICD-10 codes identified into 17 different groups, based on the nature of the lesion, which became the prediction targets of this study. The ICD-10 codes that make up each group are presented in Table S1 of this document. Table 1 (manuscript) shows the injury category, the referral made for the diagnosis and, finally, the type of priority needed for each injury.

**S2.3 Tools used**

The tools used to carry out this project and the reasons why they were selected are described in this subsection. Python was used as a programming language, due to the availability of an extensive library of open source data analysis tools, web frameworks and test instruments, offering an inviting ecosystem for programming, especially in the context of convolutional neural networks, processing of imaging and data science. For classification experiments, Keras and Tensorflow were used. Keras was designed to be an API (Application Programming Interface) adhering to the best practices of the application of artificial intelligence and reducing the cognitive load required for its use. Additionally, it offers consistent and simple APIs, minimizing the number of actions required for common use cases and providing clear feedback to users in case of errors during their execution. TensorFlow is a scalable, open source machine learning system capable of operating in heterogeneous environments. TensorFlow's proposal is to facilitate the application of the various artificial intelligence algorithms that make up its libraries, providing its users with a simple abstraction for using the algorithms based on the data flow during the training process^1^.

**S3 Exploratory experiments**

In this work, some of the main architectures of deep neural networks, the benefits of using different learning transfer paradigms and the impact of parameter variation on the accuracy of the artificial neural network were evaluated. Neural networks were applied to the classification of skin lesions with the aim of determining the correct referral for the patient's treatment, the priority of treatment and providing the physician with a diagnostic suggestion. The initial hypothesis is that deep neural networks are able to perform this classification independently, only using the images and their respective labels in a supervised learning approach without the need to apply other techniques to segment the images or assist in the extraction of features.

**S3.1 First Exploratory Experiment**

The first experiment used 1,000 images from the acquisition process dataset. In order to prevent the data set used from becoming unbalanced in view of the disparity in the amount of images in each group, the same number of images in each of the five groups was used for training and validation. The images used belong to five groups with the highest volume of diagnoses (200 images from each group). The five groups used in the experiment account for 65% of the entire volume of diagnoses present in the 17 groups (Table 1 manuscript). To assess the performance of the network, 200 images were used for validation (40 from each group). The choice of images was random, the only selection bias was the group to which the lesion belongs. For training, the momentum rate was used with a value of 0.9 according to the original configuration of the VGG, ResNet and GoogLeNet architectures. For the Learning Rate (LR), the values ​​referring to the best performance of each architecture applied to the context of classification of dermatological lesions in the literature were used.

For the ResNet architecture, LR was initialized to 10-1 and its value was divided by 10 for every 60 X 104 interactions with the training set^2^ (HAN et al, 2018). In scenarios with the VGG architecture, the learning rate was initialized with a value of 10-2 and divided by 10 whenever the accuracy of the validation set was stabilized over the training periods^3^. The GoogLenet architecture was initialized with a Learning Rate of 10-2 with a 4% decrement in its value every 8 epochs.

The images from the dataset were processed in groups of 32 images simultaneously. The training was carried out over 500 epochs.

In this experiment, 9 scenarios that constitute variations of architecture and initialization strategy of weights of deep neural networks were evaluated. The VGG, GoogLeNet and ResNet architectures with weight initialization approaches such as fixed feature extractors (FFE), extension of the model and reinitialization of weights (EMRW) and random initialization (RI) were evaluated. For learning transfer, the weights from the training of the ImageNet dataset will be used to train the architectures in the ILSVRC competition ^4, 5^. All models were trained and tested with Keras API using Tensorflow as backend on two Amazon instances: p2.xlarge with a single Nvidia Tesla k80 GPU, g3.4xlarge with a single Nvidia Tesla M60 GPU. The scenarios evaluated in the first experiment are presented in Table S3.1.

**Table S3.1 – Scenarios for the first exploratory experiment**

| Scenario | Architecture | Weights initialization strategy | LR |
| --- | --- | --- | --- |
| 1 | VGG | FFE | 10^-2^ |
| 2 | VGG | EMRW | 10^-2^ |
| 3 | VGG | RI | 10^-2^ |
| 4 | GoogLeNet | FFE | 10^-2^ |
| 5 | GoogLeNet | EMRW | 10^-2^ |
| 6 | GoogLeNet | RI | 10^-2^ |
| 7 | ResNet | FFE | 10^-1^ |
| 8 | ResNet | EMRW | 10^-1^ |
| 9 | ResNet | RI | 10^-1^ |

LR: learning rate

FFE: fixed feature extractors

EMRW: extension of the model and reinitialization of weights

RI: random initialization

**S3.2 Second Experiment**

The second experiment used the same 1,000 images as the first experiment. For training, the momentum rate with a value of 0.9 was used. However, LR was set at 10^-6^ for all architectures. The images from the dataset were processed in groups of 32 images simultaneously. The training was carried out over 500 epochs.

In this experiment, 9 scenarios that constitute variations of architecture and initialization strategy of weights of deep neural networks were evaluated. The VGG, GoogLeNet and ResNet architectures with weight initialization approaches such as fixed feature extractors (FFE), extension of the model and reinitialization of weights (EMRW) and random initialization (RI) were evaluated. For learning transfer, the weights from the training of the ImageNet dataset will be used to train the architectures in the ILSVRC competition^4, 5^. All models were trained and tested with Keras API using Tensorflow as backend on two Amazon instances: p2.xlarge with a single Nvidia Tesla k80 GPU, g3.4xlarge with a single Nvidia Tesla M60 GPU. The scenarios evaluated in the first experiment are presented in Table S3.2

**Table S3.2 – Scenarios for the second experiment**

| Scenario | Architecture | Weights initialization strategy | LR |
| --- | --- | --- | --- |
| 1 | VGG | FFE | 10^-6^ |
| 2 | VGG | EMRW | 10^-6^ |
| 3 | VGG | IR | 10^-6^ |
| 4 | GoogLeNet | FFE | 10^-6^ |
| 5 | GoogLeNet | EMRW | 10^-6^ |
| 6 | GoogLeNet | RI | 10^-6^ |
| 7 | ResNet | FFE | 10^-6^ |
| 8 | ResNet | EMRW | 10^-6^ |
| 9 | ResNet | RI | 10^-6^ |

LR: learning rate

FFE: fixed feature extractors

EMRW: extension of the model and reinitialization of weights

RI: random initialization

.

**References:**

1. ABADI, M., BARHAM P, et al. TensorFlow:

A system for large-scale machine learning. presented at: Symposium

on Operating Systems Design and Implementation; 2016; Savannah.

2. Han SS, Kim MS, Lim W, Park GH, Park I, Chang SE. Classification of the Clinical Images for Benign and Malignant Cutaneous Tumors Using a Deep Learning Algorithm. *J Invest Dermatol*. 07 2018;138(7):1529-1538. doi:10.1016/j.jid.2018.01.028

3. MAHBOD, A., SCHAEFER G, WANG C, ECKER R, ELLINGER I. Skin

Lesion Classification Using Hybrid Deep Neural Networks. 2017;

4. SIMONYAN, K., ZISSERMAN A. Very Deep Convolutional Networks for Large-Scale Image Recognition. presented at: Computer Vision and Pattern Recognition (csCV); 2014; arXiv:1409.1556 [cs.CV]

5. He K, Zhang X, Ren S, Sun J. Deep residual learning for image recognition. presented at: Conference on Computer Vision and Pattern Recognition; 2016;
